# Deciphering the Association of Cytokines, Chemokines, and Growth Factors in Chondrogenic Differentiation of Human Bone Marrow Mesenchymal Stem Cells Using an *ex vivo* Osteochondral Culture System

**DOI:** 10.3389/fcell.2019.00380

**Published:** 2020-01-17

**Authors:** Mohammad Alam Jafri, Gauthaman Kalamegam, Mohammed Abbas, Mohammed Al-Kaff, Farid Ahmed, Sherin Bakhashab, Mahmood Rasool, Muhammad Imran Naseer, Vasan Sinnadurai, Peter Natesan Pushparaj

**Affiliations:** ^1^Centre of Excellence in Genomic Medicine Research, King Abdulaziz University, Jeddah, Saudi Arabia; ^2^Department of Medical Laboratory Technology, Faculty of Applied Medical Sciences, King Abdulaziz University, Jeddah, Saudi Arabia; ^3^Sheikh Salem Bin Mahfouz Scientific Chair for Treatment of Osteoarthritis by Stem Cells, King Abdulaziz University, Jeddah, Saudi Arabia; ^4^Faculty of Medicine, Asian Institute of Medicine, Science and Technology University, Bedong, Malaysia; ^5^Department of Orthopaedic Surgery, Faculty of Medicine, King Abdulaziz University, Jeddah, Saudi Arabia; ^6^Department of Biochemistry, Faculty of Sciences, King Abdulaziz University, Jeddah, Saudi Arabia

**Keywords:** osteoarthritis, stem cells, cartilage, xMAP technology, cytokines, chemokines, growth factors, chondrogenesis, ingenuity pathway analysis

## Abstract

Osteoarthritis (OA) is a chronic degenerative joint disorder associated with degradation and decreased production of the extracellular matrix, eventually leading to cartilage destruction. Limited chondrocyte turnover, structural damage, and prevailing inflammatory milieu prevent efficient cartilage repair and restoration of joint function. In the present study, we evaluated the role of secreted cytokines, chemokines, and growth factors present in the culture supernatant obtained from an *ex vivo* osteochondral model of cartilage differentiation using cartilage pellets (CP), bone marrow stem cells (BM-MSCs), and/or BM-MSCs + CP. Multiplex cytokine analysis showed differential secretion of growth factors (G-CSF, GM-CSF, HGF, EGF, VEGF); chemokines (MCP-1, MIP1α, MIP1β, RANTES, Eotaxin, IP-10), pro-inflammatory cytokines (IL-1β, IL-2, IL-5, IL-6, IL-8, TNFα, IL-12, IL-15, IL-17) and anti-inflammatory cytokines (IL-4, IL-10, and IL-13) in the experimental groups compared to the control. *In silico* analyses of the role of stem cells and CP in relation to the expression of various molecules, canonical pathways and hierarchical cluster patterns were deduced using the Ingenuity Pathway Analysis (IPA) software (Qiagen, United States). The interactions of the cytokines, chemokines, and growth factors that are involved in the cartilage differentiation showed that stem cells, when used together with CP, bring about a favorable cell signaling that supports cartilage differentiation and additionally helps to attenuate inflammatory cytokines and further downstream disease-associated pro-inflammatory pathways. Hence, the autologous or allogeneic stem cells and local cartilage tissues may be used for efficient cartilage differentiation and the management of OA.

## Introduction

Osteoarthritis (OA) is a common inflammatory and degenerative disease of adult bone joints, and approximately 10–15% of the young and 60% of older people suffer from OA worldwide (Wittenauer et al., 2013). The disease is characterized by progressive cartilage degradation, subchondral sclerosis, and formation of osteophytes at joint margins leading to joint space narrowing as well as bone marrow lesions in advanced chronic OA (Rahmati et al., 2016). The patients with OA suffer from mild to severe chronic pain, which extensively reduces their efficiency and overall quality of life. The risk factors associated with OA include aging, obesity, genetic predispositions, certain occupations, and various co-morbid diseases, including diabetes, gout, septic arthritis, and rheumatoid arthritis (McWilliams et al., 2011; Eymard et al., 2015; Verbeek et al., 2017). The extracellular matrix (ECM) significantly contributes to cartilage development and maintenance of the biomechanical properties, and the OA is associated with progressive deterioration of ECM, and less cell turnover directly limits the self-repair ability of the cartilage.

Current clinical treatment of OA includes the use of analgesics and non-steroidal anti-inflammatory drugs (NSAIDs), which have some limitations for their long-term use, as they lead to gastrointestinal, renal, cardiac and neurological complications (Abraham et al., 2007). Therefore, numerous alternate approaches including bone marrow stimulating techniques such as osteochondral autologous graft (collected from the patient’s own iliac crest, tibia or fibula), allogenic graft (harvested from different patients) or synthetic graft (made of biocompatible materials) transplantations have been used to treat cartilage defects (Foldager, 2013). Autologous bone grafts are the most preferred approach for OA treatment as they significantly promote cartilage regeneration without the risk of developing infection and rejection. However, multiple arthroscopic incision sites may be needed to obtain a suitable autograft, which can increase the risk of nerve injury or damage. Allogenic grafts are also frequently used, but they carry a potential risk of immune rejection, infection, and delayed healing. Synthetic osteochondral grafts have been used for the treatment of osteochondral lesions (Pearce et al., 2012), and they provide a viable alternative option to both autologous and allogenic grafts but are still under evaluation for their large-scale clinical applications (Jia et al., 2017). Another significantly advanced approach for the healing of damaged articular cartilage is autologous chondrocyte implantation (ACI), which involves arthroscopic removal of a small piece of articular cartilage from the patient’s knee for self-repair. The removed biopsy is processed for the isolation and culture of chondrocytes to obtain the required number of cells for subsequent transplantation to the sites of the chondral defect. Initial clinical studies were encouraging, but there were certain disadvantages such as donor site injury due to removal of articular cartilage piece for chondrocyte extraction, loss of chondrocytic phenotype during culture expansion, and the procedure usually applies to more extensive lesions. While the intra-articular injection of viscous supplements (hyaluronic acid, autologous conditioned serum) is useful for the treatment of symptoms in mild to moderate knee OA, it also leads to painful side effects, including local swelling, inflammation, knee bursitis, and sometimes allergic reactions. The delivery of human stem cells to the damaged cartilage area to aid repair appears to be a better strategy for OA treatment. Several sources of human stem cells and scaffolds have been used to optimize the best human stem cell source and a suitable scaffold. The human stem cells derived from umbilical cord Wharton’s jelly grown on collagen-nano scaffolds have been proposed for the treatment of OA (Fong et al., 2012; Gauthaman et al., 2013). However, delivery of autologous bone marrow-derived mesenchymal stem cells (BM-MSCs) in a suitable vehicle by direct intra-articular injection may be a promising approach for the OA treatment, and also this is less invasive than open arthroplasty (Wang et al., 2015). Autologous or allogeneic MSCs isolated from bone marrow have also been implanted to facilitate regeneration of affected articular cartilage (Lee and Wang, 2017). MSCs, along with their differentiation ability, also demonstrate immunosuppressive properties mediated *via* secretion of a variety of soluble factors, including anti-inflammatory cytokines (Zhao et al., 2016). These essential characteristics of MSCs make them highly suitable for allogeneic cell therapy in OA.

To understand the possible role of cytokines in OA and its management, herein, we evaluated the secretory factors from an *ex vivo* osteochondral model, which was used in chondrogenic differentiation of the human bone marrow mesenchymal stem cells (BM-MSCs) for cartilage tissue regeneration.

## Results

**FIGURE 1:**
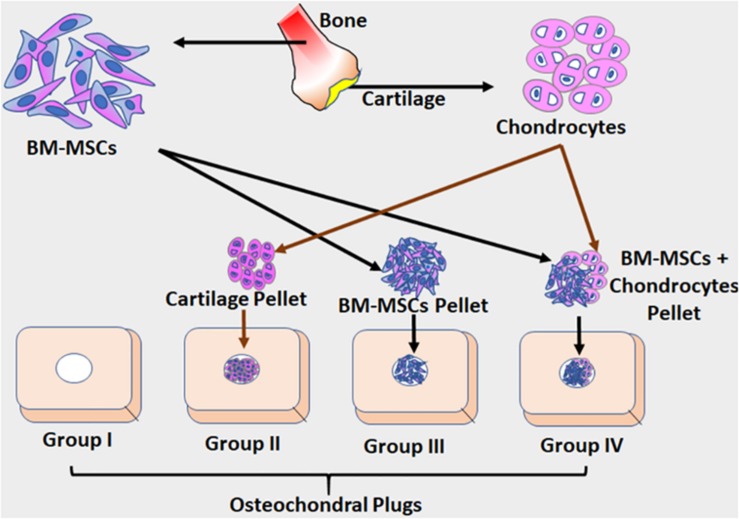
The schematic illustration of the *ex vivo* osteochondral model adopted in this study. Osteochondral plugs were obtained from patients undergoing total knee arthroplasty, and a central drill defect (2 mm) was made to simulate full-thickness cartilage damage. Cartilage shavings from healthy cartilage were homogenized to obtain cartilage pellets (CP). Three different groups were used in experiments. Group I – the osteochondral plug alone (control); Group II – the osteochondral plug filled with homogenized cartilage pellet (1.0 mm); Group III – the osteochondral plug filled BM-MSCs (1 × 10^6^ cells) as a cell pellet and Group IV – the osteochondral plug filled with BM-MSCs (0.5 × 10^6^ cells) and homogenized cartilage pellets (0.5 mm). Both control and the experimental groups were cultured using chondrogenic differentiation media (Lonza) for 28 days under standard culture conditions, with media additions every 72 h. The culture supernatants collected at D28 from both control and experimental groups were evaluated for secreted cytokines using Luminex based xMAP assay.

**FIGURE 2:**
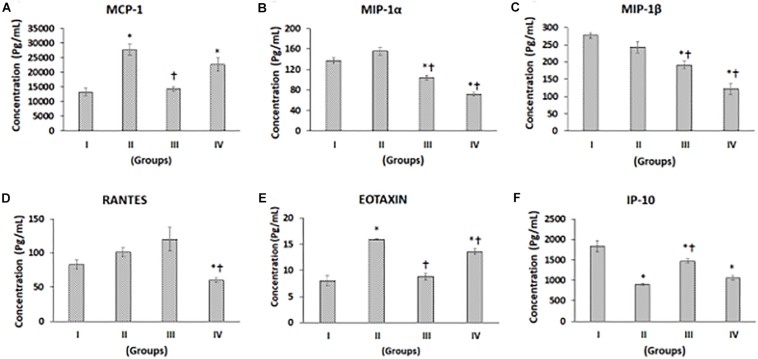
Luminex xMAP assay for chemokines. The culture supernatants collected at D28 from both control and experimental groups were evaluated for secreted chemokines such as **(A)** MCP-1, **(B)** MIP-1a, **(C)** MIP-1b, **(D)** RANTES, **(E)** Eotaxin, and **(F)** IP-10. The secreted concentrations (pg/mL) of MCP-1, MIP-1α, MIP-1β, RANTES, Eotaxin, and IP-10 are expressed as mean ± SD. ^∗^*P* ≤ 0.05 compared to control group I and ^†^*P* ≤ 0.05 compared to group II was considered to be statistically significant.

**FIGURE 3:**
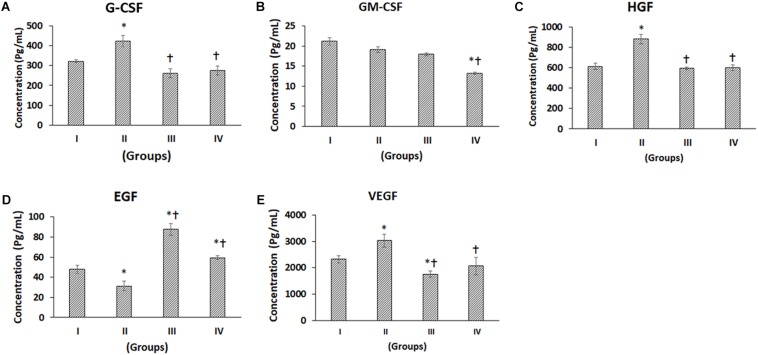
Luminex xMAP assay for growth factors. The culture supernatants collected at D28 from both control and experimental groups were evaluated for secreted Growth Factors such as **(A)** G-CSF, **(B)** GM-CSF, **(C)** HGF, **(D)** EGF, and **(E)** VEGF. The secreted concentrations (pg/mL) of G-CSF, GM-CSF, HGF, EGF, and VEGF are expressed as mean ± SD. ^∗^*P* ≤ 0.05 compared to control group I and ^†^*P* ≤ 0.05 compared to group II was considered to be statistically significant.

**FIGURE 4:**
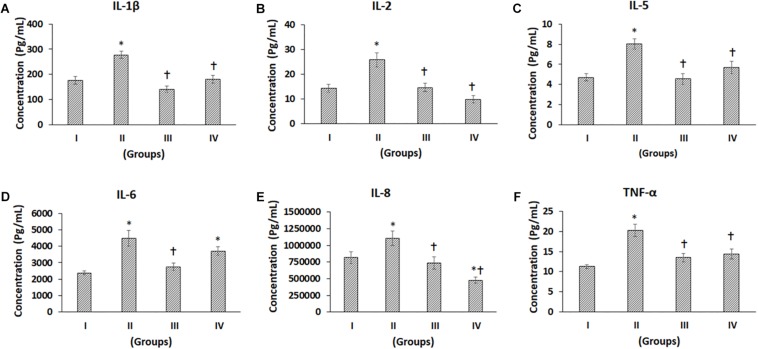
Luminex xMAP assay for proinflammatory cytokines. The culture supernatants collected at D28 from both control and experimental groups were evaluated for secreted proinflammatory cytokines such as **(A)** IL-1b, **(B)** IL-2, **(C)** IL-5, **(D)** IL-6, **(E)** IL-8, and **(F)** TNF-a. The secreted concentrations (pg/mL) of IL-1b, IL-2, IL-5, IL-6, IL-8, and TNF-a are expressed as mean ± SD. ^∗^*P* ≤ 0.05 compared to the control group I and ^†^*P* ≤ 0.05 compared to group II was considered to be statistically significant.

**FIGURE 5:**
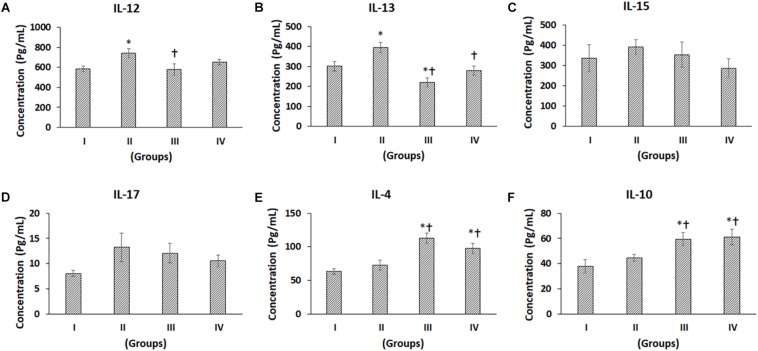
Luminex xMAP assay for proinflammatory and anti-inflammatory cytokines. The culture supernatants collected at D28 from both control and experimental groups were evaluated for secreted cytokines such as **(A)** IL-12, **(B)** IL-13, **(C)** IL-15, **(D)** IL-17, **(E)** IL-4, and **(F)** IL10. Data were expressed as mean ± SD. ^∗^*P* ≤ 0.05 compared to the control group I and ^†^*P* ≤ 0.05 compared to group II was considered to be statistically significant.

**FIGURE 6:**
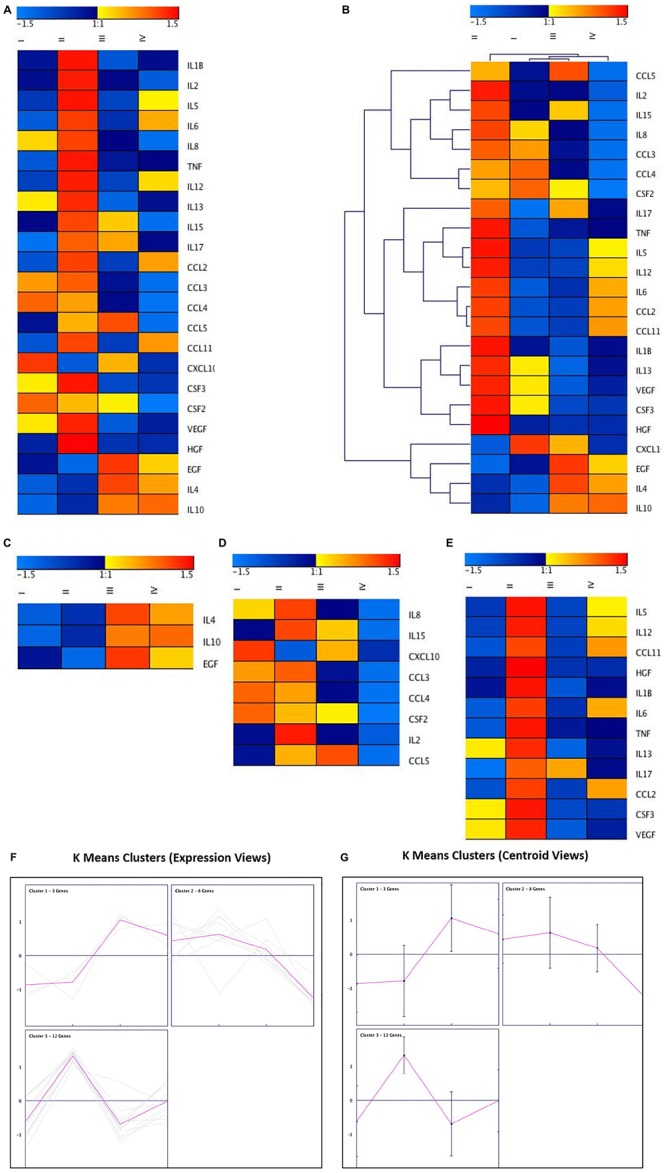
Cluster analysis of cytokines, chemokines, and growth factors using genesis software. **(A)** Heatmap of the differentially regulated cytokines, chemokines, and growth factors in *ex vivo* Control (I), *ex vivo* + MSCs (II), *ex vivo* + Cartilage + MSCs groups (III and IV). **(B)** Hierarchical Clustering of the differentially regulated cytokines, chemokines and growth factors in *ex vivo* Control, *ex vivo* + MSCs and *ex vivo* + Cartilage + MSCs groups **(C–E)** K-Means clustering of the differentially regulated cytokines, chemokines and growth factors in *ex vivo* Control, *ex vivo* + MSCs and *ex vivo* + Cartilage + MSCs groups with both **(F)** Cytokine Secretion and **(G)** Centroid views of all the three K-Means Clusters obtained using Genesis Software.

**FIGURE 7:**
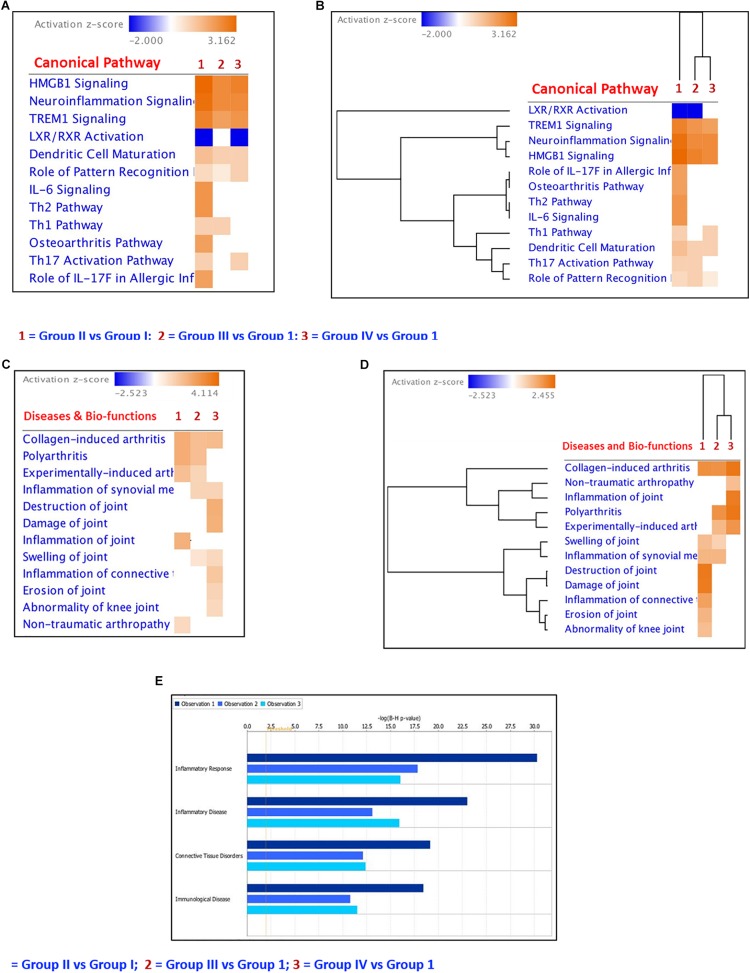
Ingenuity Pathway core analyses of the differentially regulated cytokines, chemokines and growth factors. **(A)** Heatmap of the differentially regulated Canonical Pathways in *ex vivo* + MSCs and *ex vivo* + cartilage + MSCs groups; **(B)** Hierarchical clustering of the differentially regulated canonical pathways in *ex vivo* + MSCs and *ex vivo* + Cartilage + MSCs groups compared to the *ex vivo* control group; **(C)** Heatmap of the differentially regulated diseases and bio-functions related to connective tissue disorders in *ex vivo* + MSCs and *ex vivo* + Cartilage + MSCs groups; **(D)** Hierarchical clustering of the differentially regulated diseases and bio-functions related to connective tissue disorders in *ex vivo* + MSCs and *ex vivo* + Cartilage + MSCs groups compared to the *ex vivo* control group; **(E)** Bar graphs showing the regulation of inflammatory response, inflammatory disease, connective tissue disorders, and immunological diseases by the differentially expressed cytokines, chemokines, and growth factors in the experimental groups compared to the control group. The dark blue color bar (Observation 1) shows Group II (CP) vs. Group I (control) comparison; the light blue bar represents Group III (BM-MSCs) vs. Group I (control) comparison (Observation 2) and the cadet blue bar shows Group IV (BM-MSCs + CP) vs. Group I (control) comparison (Observation 3).

BM-MSCs progenitors adhered well to the culture surface and demonstrated active proliferation. The cells reached 70–80% confluence within 14 days and were expanded in sub-cultures. The BM-MSCs showed characteristic spindle-shaped morphology, expressed MSC related CD markers and underwent differentiation into chondrocytes in the osteochondral explant model system used for the evaluation of chondrogenic differentiation of the human bone marrow mesenchymal stem cells (Abbas, 2017). Multiplex Luminex bead-based analysis of the cell culture supernatant revealed differential expression of various cytokines, chemokines, and growth factors in the experimental groups, and these results are provided in the form of different figures and heat maps (Figures 1–7).

### Multiplex Luminex xMAP Assay of Chemokines

Chemokines such as monocyte chemoattractant protein-1 (MCP-1), macrophage inhibitory protein-alpha (MIP-α), macrophage inhibitory protein-beta (MIP-β), regulated on activation, normal T cell expressed and secreted (RANTES, C-C chemokines), Eotaxin, and interferon gamma-induced protein 10 (IP10), a C-X-C chemokine, levels were measured in supernatants from control, and experimental groups showed differential secretion pattern upon chondrogenic differentiation (Figures 2A–F). MCP-1 levels were higher in Group II (CP) and Group IV (BM-MSCs + CP), while it was decreased in Group III (BM-MSCs) compared to Group I (control). The increases in MCP-1 by 107.95 and 69.90% in Group II and Group IV, respectively, were statistically significant compared to the control (Group I) (Figure 2A). Also, compared to Group II (CP), both Groups III and IV demonstrated decreases in MCP-1 levels, and the reduction in Group III by 47.90% was statistically significant (Figure 2A). MIP-1α showed a mild increase in Group II by 13.43% and significant decreases in Group III and Group IV by 24.13 and 48.18% compared to control (Group I) (Figure 2B). Besides, compared to Group II (CP), both Groups III and IV demonstrated significant decreases by 33.11 and 54.31%, respectively (Figure 2B). MIP-1β showed reductions by 12.23, 30.93, and 56.03% in Groups II, III, and IV, respectively, compared to control (Group I). Of these, the reductions observed in Group III and IV were statistically significant (Figure 2C). Also, compared to Group II (CP), both Groups III and IV demonstrated significant decreases by 21.31 and 49.90% in Group III and IV, respectively (Figure 2C). The levels of RANTES were increased in Group II by 21.91% and Group III by 44.71%, while it was significantly decreased in Group IV by 27.62% compared to Group I (Figure 2D). Also, compared to Group II, RANTES showed a significant decrease in Group IV by 40.63% (Figure 2D). Eotaxin levels were significantly increased in Groups II and IV by 99.0 and 69.41%, respectively (Figure 2E). Compared to Group II, Eotaxin was significantly decreased in Groups III and IV by 44.92 and 14.87%, respectively (Figure 2E). IP-10 levels were reduced in Groups II, III, and IV by 51.31, 20.74, and 42.55%, respectively, and all these decreases were statistically significant (Figure 2F). Compared to Group II, the levels of IP-10 was significantly increased in Group III (by 62.78%) and mildly increased in Group IV by 19.99% (Figure 2F).

### Multiplex Luminex xMAP Assay of Growth Factors

The following growth factors, namely granulocyte colony-stimulating factor (G-CSF), granulocyte-macrophage colony-stimulating factor (GM-CSF), hepatocyte growth factor (HGF), epidermal growth factor (EGF) and vascular endothelial growth factor (VEGF) in general demonstrated differential secretion following chondrogenic differentiation (Figures 3A–E). G-CSF was significantly increased in Group II (CP) by 31.41% compared to Group I (control), while Group III (BM-MSCs) and Group IV (BM-MSCs + CP) were decreased by 18.82 and 14.66% respectively (Figure 3A). Also, compared to Group II, both Groups III and IV were significantly reduced by 38.23 and 35.06%, respectively (Figure 3A). GM-CSF was decreased in Groups II, III, and IV by 9.65, 15.33, and 37.37% respectively compared to the control (Group I). Of these, only the decrease in Group IV was statistically significant Also, compared to Group II, Group IV showed a significant reduction by 30.68% (Figure 3B). The levels of secreted HGF was significantly increased in Group II by 43.49% compared to the control (Group I). Also, compared to Group II, the HGF levels were significantly decreased in Groups III and IV by 32.67 and 31.77%, respectively (Figure 3C). EGF demonstrated a significant decrease in Group II by 34.70% and significant increases in Groups III and IV by 82.71 and 23.92%, respectively, compared to the control (Group I). Besides, compared to Group II, the EGF levels were significantly increased in Groups III and IV by 179.79 and 89.75%, respectively (Figure 3C). VEGF showed a significant increase in Group II (by 30.53%) and a significant decrease in Group III (by 24.34%) compared to the control (Group I). Also, compared to Group II, the VEGF levels were significantly decreased in Groups III and IV by 42.04 and 31.79%, respectively (Figure 3E).

### Multiplex Luminex xMAP Assay of Pro-inflammatory and Anti-inflammatory Cytokines

The pro-inflammatory cytokines such as IL-1β, IL-2, IL-5, IL-6, IL-8, TNF-α, IL-12, IL-15, and IL-17 showed differential secretion patterns compared to the Control (Group I) (Figures 4A–F, 5A–D). IL-1β showed a significant increase in Group II by 57.82% compared to the control (Group I), while Group III showed a mild decrease, and Group IV demonstrated a similar level of IL-1β expression as control (Group I). However, when compared with Group II, both Groups III and IV showed significant decreases by 49.11 and 34.99%, respectively (Figure 4A). IL-2 showed a significant increase in Group II by 81.61% compared to Control (Group I). Compared to Group II, both Groups III and IV showed significant decreases by 43.35 and 61.82%, respectively (Figure 4B). IL-5 showed a significant increase in Group II by 71.07% compared to the control (Group I). However, compared with Group II, both Groups III and IV showed significant decreases by 43.55 and 29.16%, respectively (Figure 4C). The levels of IL-6 were significantly increased in Group II and Group IV by 87.41 and 55.77%, respectively, compared to control (Group I). Compared to Group II, there was a significant decrease in Group III by 38.25% (Figure 4D). IL-8 levels were increased in Group II by 35.18% and decreased in Groups III and IV by 13.4 and 41.72%, respectively. Also, compared to Group II, IL-8 was significantly decreased in Groups III and IV by 33.34 and 56.88% (Figure 4E). TNF-α demonstrated an increase in Groups II, III and IV by 79.72, 19.57, and 26.84%, respectively, compared to the control (Group I). Of these, only the increase in Group II was significant. Also, compared to Group II, TNF-α was significantly decreased in Group III and Group IV by a value of 33.46 and 29.42%, respectively (Figure 4F). IL-4 demonstrated increases in Groups II, III, and IV by 14.43, 77.03, and 53.44%, respectively. Of these, only the improvements in Group III and Group IV were significant (Figure 5E). Also, compared to Group II, the IL-4 levels were significantly increased in Group III and Group IV by 54.70 and 34.09% (Figure 5E). The concentration of IL-12 was significantly increased in Group II by 27.69% compared to control (Group I). Also, IL-12 was significantly decreased in Group III by 22.21% compared to Group II (Figure 5A). IL-13 showed an increase in Group II by 31.20%, while it was reduced in Group III by 26.76%. Compared to Group II, both Group III and IV showed significant decreases by 44.17 and 29.21%, respectively (Figure 5B). The levels of IL-15 showed mild increases in Group II (16.61%) and Group III (5.27%), while Group IV showed a slight decrease (14.74%), and these values were not significant (Figure 5C). The levels of IL-17 showed increases in Groups II, III, and IV by 64.02, 49.88, and 30.77%, respectively, compared to control (Group I) (Figure 5D).

The anti-inflammatory cytokine, namely IL-10, showed increased levels compared to the control. IL-10 demonstrated increases in Groups II, III, and IV by 17.70, 56.74, and 61.17%, respectively. Of these, only the increases in Group III and Group IV were significant (Figure 5F). Also, compared to Group II, the IL-10 levels were significantly increased in Group III and Group IV by 33.17 and 36.93% (Figure 5F).

### Heatmap and Cluster Analyses Using Genesis

The mean cytokine secretion data were used to generate a heatmap that showed the fold changes in chemokines, growth factors the pro- and anti-inflammatory cytokines (Figure 6A). Blue and red colors indicate lower and higher secretion limits. The chemokines MIP-1α and MIP-1β showed decreased secretion in Group III. The fold changes with MCP-1 in Group III were similar to that of the control (Group I), while RANTES (CCL5) demonstrated an increase in Group III and a decrease in Group IV (Figure 6A). Of the growth factors, VEGF showed a reduction in Group III compared to both Groups I and II (Figure 6A). The pro-inflammatory cytokines (IL-8 and IL-15) were decreased in Group III, while the anti-inflammatory cytokine IL-10 was reduced in Group II and increased in Group III compared to Group I (Figure 6A). These increases or decreases in fold changes were in correlation with that of the Luminex cytokine results. Hierarchical clustering was determined based on the fold changes of the cytokines, and the inter and intra distances between various cytokines are demonstrated (Figure 6B). The K Means Clustering of the MAGPIX data revealed three significant clusters (Figures 6C,D) with IL-4, IL-10, and EGF clustering together (Figure 6C), and the remaining two clusters predominantly have the proinflammatory cytokines and growth factors (Figures 6D,E). Besides, both cytokine secretion and centroid views were generated using the Genesis Software (Figure 6G) to understand the secretory pattern of cytokines, chemokines, and growth factors in the experimental groups compared to the control group.

### Ingenuity Pathway Analysis

IPA core analysis was performed to understand the various canonical pathways regulated by the differentially secreted cytokines, chemokines, and growth factors in Group II and Group III. Using comparison analysis module in IPA, we have identified differentially regulated canonical pathways such as OA Pathway, HMGB Signaling, TREM1 Signaling, IL-6 Signaling, LXR/RXR Signaling, Th17 Activation Pathway, Role of Pattern Recognition Receptors, Th1 and Th2 Pathways, Dendritic Cell Maturation, etc. (Figures 7A,B). The heat map and hierarchical clustering showed that the OA pathway signaling, IL-6 signaling, Th1, and Th2 Pathways were decreased significantly in Group III and IV compared to Group II (Figures 7A,B). The heatmap and hierarchical clustering of the diseases and bio functions related to the connective tissue disorders showed that the inflammation of the joint and the polyarthritis was decreased in relation to BM-MSCs and cartilage + BM-MSCs compared to Group II (Figures 7C,D). Furthermore, the diseases and bio-functions related to Inflammatory Response, Inflammatory Disease, Connective Tissue Disorders, and Immunological Diseases were decreased in Group IV (CP + BM-MSCs) compared to other experimental groups (Group II and Group III) (Figure 7E) as indicated by the length of each colored bar corresponding to their comparative involvement in diseases and their potential bio-functions.

## Discussion

OA is a common disease usually affecting persons aged 60 years or older (Kotlarz et al., 2009; Alkan et al., 2014) involving progressive degeneration of cartilage and severe inflammatory response leading to chronic pain and disability. The inflammatory processes are mediated by cytokines, a group of small proteins/polypeptides involved in the inflammatory and immunological responses. It is of prime importance to understand their expression profile in OA to facilitate the successful management of OA, including cell-based therapies. The use of an *ex vivo* model system of cells-laden osteochondral plugs has provided better insights on the secretory pattern of various chemokines, growth factors, pro- and anti-inflammatory cytokines which may play a vital role in the repair of damaged articular cartilage.

The higher levels of MCP-1 secreted in Group II could be due to the presence of homogenized cartilage pellets as MCP-1 is reported to be higher in the presence of cartilage damage, a common pathological occurrence in OA (Xu et al., 2015). A combination of BM-MSCs and CP significantly reduced the levels of MCP-1, indicating that stem cells have anti-inflammatory properties. Lower levels of macrophage inflammatory proteins (MIP-1α, MIP-1β) and RANTES as observed with Groups III and IV may in fact indirectly help survival of transplanted cells, as monocytes attraction to the OA joint and the immediate adverse inflammatory response which is a common phenomenon during direct cell transplantations will be minimal or absent (Sato et al., 2012). Lower secretory levels of eotaxin-1 in Group III and IV compared to the control indicates that MSCs possess anti-inflammatory property. Increased secretion of eotaxin-1 is reported in chronic inflammatory lesions of the bone, including rheumatoid arthritis (Kokkonen et al., 2010), and is associated with osteoclast migration and bone resorption (Kindstedt et al., 2017).

Interestingly, our results are in contrast compared to an earlier study, which analyzed eotaxin-1 secretion from the serum of healthy and OA patients where healthy controls secreted higher levels of eotaxin-1, and it was almost undetectable in OA patients (Beekhuizen et al., 2013). This could be attributed to (i) the differences in the samples studied such as serum and cell culture supernatant; (ii) the influence of *in vivo* inherent physiological counter mechanisms unlike the *in vitro* system and (iii) differences in the assay method between standard and Luminex based multiplex (present study) that were used to analyze the serum and culture supernatant respectively. IP-10 was decreased in all experimental groups compared to control; however, it was increased in comparison to Group II. In line with our studies, the serum samples of healthy patients were reported to have higher levels of IP-10 than in OA (Saetan et al., 2011). IP-10 is associated with progenitor cell mobilization, which then contributes to tissue repair, and their decrease in OA is associated with disease severity (Saetan et al., 2011).

G-CSF regulates hemopoiesis and is associated with survival, the proliferation of neutrophil progenitor cells as well as the release of mesenchymal progenitors. G-CSF accelerated cartilage regeneration in an osteochondral defect model of the patellar bone in a rodent model. There was an initial recruitment of neutrophils at the defect site, which was then followed by the infiltration of MSCs/progenitors that contributed to cartilage regeneration (Okano et al., 2014). GM-CSF is commonly associated with the mobilization of hematopoietic stem cells as well as activation of dendritic cells and macrophages (Truong et al., 2017). It was also demonstrated to increase MSCs at the microfracture sites of a full-thickness chondral defect and thereby enhance cartilage repair *in vivo* in New Zealand white rabbits (Truong et al., 2017). GM-CSF promotes repair of the damaged cartilage by stimulating proliferation of MSCs (Okano et al., 2014; Sasaki et al., 2017) whereas EGF binding to its receptor activates EGFR-mediated signaling which is crucial for the maintenance of chondrocyte number by protecting them from undergoing apoptosis during OA progression (Zhang et al., 2014; Jia et al., 2016). HGF was upregulated in OA cartilage and synovial fluids of patients compared to healthy subjects (Abed et al., 2015). In our study, HGF secretion was significantly lower in osteochondral defect groups that contained MSCs (Group III and Group IV). HGF induces the release of MCP-1, which mediates entry of monocytes/macrophages into the OA joints as well as promotes fracture healing by enhancing expression of bone morphogenetic protein (BMP) receptors (Imai et al., 2005).

Additionally, HGF, along with several soluble factors including IL-10, transforming growth factor-beta -1 (TGF-β), prostaglandin E2 (PGE2), indoleamine 2, 3 dioxygenase (IDO) and inducible NO synthase (iNOS) facilitates immunosuppressive action of MSCs (26). The presence of VEGF detected in this study is supported by previous studies, which have shown that it plays a role in inflammation-induced neovascularization of cartilage during OA development (Murata et al., 2008). Therefore, VEGF may be a potential therapeutic target to develop novel treatment for OA (Hamilton et al., 2016).

We found detectable concentrations of both pro-inflammatory and anti-inflammatory cytokines in the culture supernatant, suggesting that the cytokine-mediated pathways play a significant role in the pathophysiology of OA. The following pro-inflammatory cytokines, namely IL-1β, IL-2, IL-5, IL-6, IL-8, and TNF-α were increased in the present study (Figures 4A–F). IL-1β mediates the chondrocytic release of proteolytic enzymes such as matrix metalloproteinases and nitric oxide, which is known to have inflammatory activity (Chabane et al., 2008). IL-2 levels were reported to be higher in the synovial fluid of OA patients and were directly proportional to disease severity (Vangsness et al., 2011). IL-5, IL-6 along with TNF-α was reported to be increased in early stages of OA (Mabey and Honsawek, 2015). TNF-α is well known to upregulate the collagenases leading to cartilage destruction (Salminen et al., 2002). Pro-inflammatory chemoattractant cytokine IL-8 (also known as CXCL-8) produced by chondrocytes has been demonstrated to be overexpressed in synovial fluids of OA patients compared to controls (Pierzchala et al., 2011). It promotes OA progression by stimulating several critical pathophysiological events including the release of MMP-13 and neutrophil accumulation at the site of damaged articular cartilage (Borzì et al., 2004). In our study, we detected significantly lower concentrations of IL-8 in Group III and Group IV compared to Group I (control), suggesting that autologous MSCs and cartilage pellets may be utilized to control OA progression. It has been recently reported that the extracellular vesicles secreted by BM-MSCs were internalized into the OA chondrocytes and helped in the reduction of the pro-inflammatory cytokines such as IL-1α, IL-1β, IL-6, IL-8, and IL 17 as well as the TNF-α induced collagenase activity (Vonk et al., 2018).

We also detected the presence of pro-inflammatory cytokines, namely IL-12, IL-15, and IL-17 in culture supernatants (Figures 5A,C,D). IL-12 is secreted by infiltrating macrophages and synovial lining cells and shown to be involved in the maintenance of inflammation leading to articular damage in OA (Sakkas et al., 1998). IL-13 is reported to be increased in the synovial fluid of OA patients along with periostin secretion by the synoviocytes (Ishikawa et al., 2015). In the present study, both IL-12 and IL-13 were significantly reduced in the presence of stem cells indicating the anti-inflammatory property of BM-MSCs and hence, the advantage in using them for cartilage regeneration/repair. The levels of IL-15 and IL-17 are reported to be higher in OA patients and also are associated with the severity of pain in OA patients (Sun et al., 2013; Liu et al., 2015). Higher levels of IL- 17 in the synovial fluid of OA patients correlates with the end-stage disease with the presence of osteophytes, sclerosis, and significant joint space narrowing (Snelling et al., 2017). Interestingly, the levels of IL-15 and IL-17 were mildly decreased in the presence of BM-MSCs.

The cytokines IL-4 and IL-10 are reported to have anti-inflammatory properties, and their levels were found to be significantly increased in the groups that contained BM-MSCs. Both IL-4 and IL-10 are involved in the suppression of inflammation of the synovial membrane, slow down the progression of OA, and promote cartilage turnover (Mabey and Honsawek, 2015). The anti-inflammatory property does not depend directly on the MSCs but rather on the inflammatory status of the chondrocytes or synoviocytes and the local milieu to bring about the anti-inflammatory effect by acting on molecules and pathways downstream (Manferdini et al., 2013).

Heat map analyses were done using mean cytokine expression values following treatment with BM-MSCs, and BM-MSCs + cartilage demonstrated lower levels of pro-inflammatory cytokines, chemokines, and the growth factors. The canonical pathways and hierarchical clustering analysis indicated that treatment with BM-MSCs and BM-MSCs + cartilage was effective in decreasing both the secretion of several cytokines and growth factors as well as the signaling pathways involved in the progression of OA. Fischer’s test in IPA helps to identify the bio functions and canonical pathways that are significantly associated with the cytokines of interest to the disease. Most of the identified pathways following stem cells’ interaction in the management of OA were in line with an earlier study report (Ning et al., 2016), which included cytokines, p38 MAPK, and TNF receptors signaling (Ning et al., 2016).

The treatment of OA is still a challenge as the main most therapeutic measures help only in relieving pain and controlling inflammation rather than repairing damaged cartilage. Current OA treatment includes the use of non-steroidal anti-inflammatory drugs (NSAIDs), opioids, intraarticular corticosteroids, and other slow-acting symptomatic drugs such as glucosamine sulfate, chondroitin sulfate, and intraarticular hyaluronic acid (Hermann et al., 2018). However, none of these treatments are able to provide permanent benefit to OA patients. Therefore, novel therapies based on disease-modifying efficacy are urgently needed for OA patients. The direct transfer of *in vitro* differentiated chondrocytes or mesenchymal progenitors using suitable cell delivery matrices in both preclinical and clinical studies have shown improvement in cartilage repair (Freitag et al., 2016). In a clinical trial study, a single intra-articular injection of *in vitro* expanded bone marrow-derived MSCs was found to be effective in improving functional status in all patients and resulted in increased cartilage thickness in 3 out of 6 patients (Emadedin et al., 2012). The *ex vivo* osteochondral defect repair model, as used in the present study helps us in identifying the molecules that mediate inflammation in OA as well as the growth factors, chemokines, and cytokines, which directed both stem cells and native cartilage pellet toward cartilage differentiation. The insights obtained from the present study on differential expression of chemokines, cytokines and growth factors involved in cartilage tissue regeneration will provide decision-making and support in formulating a better treatment strategy for osteoarthritis. Additionally, it will further help us to select potential molecules for their evaluation in pre-clinical *in vivo* studies to develop superior diagnostic and treatment options for osteoarthritis.

However, certain limitations that we faced while conducting the present study should be noted to treat OA patients. Though the Luminex based multiplex cytokine analysis system is robust, we were unable to detect all the cytokines from the panel used, which could be due to their complete absence or minimal expression that falls below the detection limits. In the present study, we had used BM-MSCs harvested and expanded from OA patients that by itself could have contributed to the observed cytokine pattern, even though it recapitulates autologous stem cell transplantation. In future, we aim to study the cytokine profiles of MSCs derived from different sources at a relatively young age to further understand the possibilities of transplantation using allogeneic cells for cartilage tissue regeneration.

## Materials and Methods

### Ethical Approval

The Ethical Committee for Scientific Research of King Abdulaziz University, Jeddah, Saudi Arabia, had approved our protocol (11–557) used in this study. Informed consent was taken from all patients undergoing total knee arthroplasty procedure at the department of orthopedic surgery at the King Abdulaziz University Hospital (KAUH), before the collection of bone marrow aspirates, osteochondral plugs, and cartilage from undamaged areas from six OA patients.

### Sample Collection and BM-MSCs Isolation

The bone marrow aspirates were collected from OA patients undergoing knee surgery in heparinized test tubes (Becton Dickinson, Franklin Lakes, NJ, United States). BM-MSCs were established in culture using the earlier published protocol (Leyh et al., 2014; Abbas, 2017). Briefly, the bone marrow aspirate (2 ml) were directly plated in a T-175 cm^2^ flask (Greiner Bio-one) containing Dulbecco’s Modified Eagles Medium (DMEM; Sigma Aldrich, St. Louis, MO, United States) supplemented with 10% (v/v) gamma-irradiated fetal bovine serum (Sigma Aldrich), 2 mM GlutaMax (Invitrogen, Thermo Fisher Scientific, Waltham, MA, United States) and antibiotic solution [penicillin (100 IU/ml; streptomycin (100 μg/ml); Sigma Aldrich]. The adherent cells were sub-cultured and expanded under standard culture conditions of 37°C and 5% CO_2_ and maintained in an incubator.

### Preparation of Cartilage Pellet

The cartilage shavings were prepared according to the method as described by Abbas (2017). The cartilage shavings were obtained from the healthy areas of the cartilage from within the knee joints in patients undergoing total knee arthroplasty. They were washed thrice in PBS and homogenized using a tissue homogenizer under sterile conditions. The cartilage tissue homogenate was washed thrice in PBS and centrifuged to remove the flocculent tissue debris. The homogenized cartilage pellet (CP) was aliquoted to obtain approximately 0.5 mm sized pellets for subsequent use in experiments.

### Osteochondral Defect Model for Cartilage Regeneration

Osteochondral bone pieces were obtained from patients undergoing total knee arthroplasty, and a central drill defect (2 mm) was made to simulate full-thickness cartilage damage and used as an *ex vivo* model to study cartilage repair as described before (Abbas, 2017) (Figure 1). Briefly, the osteochondral plugs with central drill defect were divided into four different groups and evaluated as follows: Group I – the osteochondral plug alone (control); Group II – the osteochondral plug filled with homogenized cartilage pellet (1.0 mm); Group III – the osteochondral plug filled with BM-MSCs (1 × 10^6^ cells) as a cell pellet and Group IV – the osteochondral plug filled with BM-MSCs (0.5 × 10^6^cells) and homogenized cartilage pellets (0.5 mm). Both control (*n* = 3) and the experimental groups (*n* = 3) were cultured using chondrogenic differentiation media (Lonza, Basel, Switzerland) for 28 days under standard culture conditions, with media additions every 72–96 h. The culture supernatants collected on the 28th day (D28) from both control and experimental groups were evaluated for secreted cytokines using Luminex based multiplex cytokine assay.

### Luminex xMAP Assay for Cytokines, Chemokines, and Growth Factors

The cytokines, chemokines, and growth factor levels in culture supernatant were measured using Human Cytokine Magnetic 30-Plex Panel (Novex^®^ Invitrogen, Thermo Fisher Scientific, Waltham, MA, United States) according to the manufacturer’s protocol. Briefly, 25 μl of antibody-containing magnetic beads for 30 different analytes were added to the assay wells of Mylar plate and washed twice with 1X wash buffer. Then 50 μl of incubation buffer (provided with assay kit) was transferred into each assay well. Freshly prepared standard solutions (1:3 serial dilution) and samples (undiluted culture supernatant) were prepared and added (100 μl) to the beads. The assay plate was incubated in an orbital shaker at 500 rpm for 2 h to enable the capture of the analytes. Following incubation, the secondary antibodies were added to assay plate and incubated for 1 h. Then streptavidin-RPE coupled detection antibodies were added, and the plate was incubated for 30 min, followed by three washings and resuspension in wash buffer. The assay plate was then analyzed using the MAGPIX^®^ instrument (Luminex Corporation, Austin, TX, United States).

### Ingenuity Pathway Analysis (IPA)

The differential expression of cytokines, chemokines, and growth factors calculated based on the fold change between the control and the experimental groups was used as an input for the Ingenuity Pathway Analysis (IPA) (Qiagen, United States). The core analysis module in IPA was used to deduce differentially regulated canonical pathways, upstream regulators, diseases and bio functions, and novel gene networks based on Fisher Exact Test (*p*-value cut off at 0.05). Besides, the comparison analysis module in IPA was used to deduce the differentially regulated canonical pathways and diseases and bio functions in the experimental groups compared to the control. The heatmaps of differentially regulated canonical pathways and diseases and bio functions related to the connective tissue disorders were generated using the IPA. The heatmaps, hierarchical clusters, and K Means Clusters of differentially regulated cytokines, chemokines, and growth factors were created using the Hierarchical Clustering Algorithm with the help of Genesis Software (Genesis 1.8.1) (Sturn et al., 2002).

### Statistical Analyses

The raw data obtained from three replicates for individual analytes were analyzed by the Luminex xPONENT multiplex assay analysis software (Luminex Corporation, Austin, TX, United States) to calculate the absolute concentration in both control and experimental groups. Additionally, the level of each analyte calculated was further analyzed using GraphPad Prism Version 7 (GraphPad Software, San Diego, CA, United States) to compute the statistical significance using Student’s unpaired *t*-test. *p* ≤ 0.05 were considered to be statistically significant.

## Data Availability Statement

The datasets generated for this study are available on request to the corresponding author.

## Ethics Statement

The studies involving human participants were reviewed and approved by King Abdulaziz Hospital Ethical Committee. The patients/participants provided their written informed consent to participate in this study.

## Author Contributions

GK, MJ, PP, MA, and MA-K designed the experiments. GK, PP, and MJ conducted the experiments. GK, MA, MA-K, MJ, FA, VS, MR, MN, SB, and PP analyzed the data. GK, MJ, PP, MA, SB, and MA-K wrote the manuscript. GK, MJ, MA, MA-K, and PP proposed the research idea. All authors contributed to the editing of the manuscript and the scientific discussions.

## Conflict of Interest

The authors declare that the research was conducted in the absence of any commercial or financial relationships that could be construed as a potential conflict of interest. The handling Editor declared shared affiliations with several of the authors and a past co-authorship and with the authors MR and MN.
